# Plain Talks on Electricity and Batteries

**Published:** 1891-09

**Authors:** 


Plain Talks on Electricity and Batteries, with Therapeutic Index. For General Practitioners and Students of Medicine, By Horatio R. Bigelow, M. D., Permanent Member of the American Medical Association; Fellow of the British Gynecological Association; Member of the Philadelphia Electro-Therapeutic Society, etc. 12mo, pp. vi.—85. Philadelphia: P. Blakiston, Son & Company. 1891.

There is one comfort about this little volume, and that is its brevity. It seems to us, however, that it tells pretty nearly all there is to be told on the subject of which it treats. In that sense it is useful, as it gives the definitions of, and describes the various questions of electricity, together with the office paraphernalia necessary to their employment. It has the usual number of illustrations of different batteries with the patentee's name prominently engraved upon them. This is all very well, and quite in keeping with the work, but we are at a loss to know what can be learned from the handsome cut on page seventy-five. The publishers have printed the book in an excellent manner.

